# QTL mapping of oleic acid content in modern VNIIMK sunflower (*Helianthus annuus* L.) lines by using GBS-based SNP map

**DOI:** 10.1371/journal.pone.0288772

**Published:** 2023-10-04

**Authors:** Rim Gubaev, Stepan Boldyrev, Elena Martynova, Alina Chernova, Tatyana Kovalenko, Yuliya Chebanova, Tatyana Peretyagina, Svetlana Goryunova, Denis Goryunov, Zhanna Mukhina, Cecile Ben, Laurent Gentzbittel, Philipp Khaitovich, Yakov Demurin

**Affiliations:** 1 Skolkovo Institute of Science and Technology, Bolshoy Moscow, Russia; 2 LLC "Oil Gene", Moscow, Russia; 3 Pustovoit All-Russia Research Institute of Oil Crops, Krasnodar, Russia; 4 FSBSI Lorch Potato Research Institute, Kraskovo, Russia; 5 Institute of General Genetics, Russian Academy of Science, Moscow, Russia; 6 Belozersky Institute of Physico-Chemical Biology, Lomonosov Moscow State University, Moscow, Russia; 7 All-Russia Rice Research Institute, Krasnodar, Russia; Jeju National University, REPUBLIC OF KOREA

## Abstract

Oleic acid is a monounsaturated fatty acid increasing oil oxidative stability. High content of oleic acid is thus a valuable trait in oilseed crops. Sunflower (*Helianthus annuus* L.) normally accumulates linoleic acid as a major fatty acid, but a mutant expressing a high oleic phenotype form was previously obtained by chemical mutagenesis and mapped on the sunflower genome. Several studies suggest the presence of additional genes involved in the control of the high content of oleic acid, with their expression possibly depending on the genetic background. To test this hypothesis, we performed a QTL mapping of the high oleic acid trait within two independent F_2_ crosses involving lines with contrasting oleic acid content from the Pustovoit All-Russia Research Institute of Oil Crops (VNIIMK) collection. We applied genotyping-by-sequencing (GBS) to construct single nucleotide polymorphism-based genetic maps and performed QTL mapping using quantitative and qualitative encoding for oleic acid content. Our results support the finding that the oleic acid content in the assessed crosses is controlled by one major effect locus. However, different dominant/recessive effects of the major locus were reported for both crosses. Additionally, a possible translocation between chromosome 7 and 14 was reported in one assessed cross. We defined a set of single nucleotide polymorphism markers for each cross which could be used for marker-assisted selection.

## Introduction

Oleic acid is the monounsaturated fatty acid that on the one hand protects the lipids against thermooxidation during frying and on the other hand has positive health effects, namely lowering cholesterol and reducing inflammation [1]. Thus many breeding efforts in oilseeds including sunflower are made with the aim of increasing oleic acid content (OAC), especially in varieties used for frying oil production [2–4]. It also should be noted that the antioxidant properties of oleic acid are increased in oils with high content of γ- and/or δ-tocopherols [5]. Thus the breeding efforts for the development of plants with increased oil resistance to thermal oxidation may target both traits [6,7].

In sunflower low natural diversity in oleic acid content was reported and sunflower typically produces amounts of oleic acid of around 20% [8]. The first sunflower variety with high oleic acid content called "Pervenets" was obtained by chemical mutagenesis [9]. Lately, this mutation was introduced by back-cross within various collections with the aim of obtaining high-oleic varieties. It was first assumed that the high oleic trait is controlled by a single dominant *Ol* gene [10]. Studies based on allelic tests suggested more complex trait inheritance. It was proposed that this trait could be controlled by three dominant complementary genes (*Ol1*-*Ol3*) and one modification gene [11,12]. RAPD markers linked with the major *Ol* gene [2] were later mapped to chromosome 14 [13]. *Ol* gene explains up to 56% of phenotype variance, and was demonstrated to be the FAD2-1 gene encoding fatty acid desaturase that converts oleic acid to linoleic acid [14].

Additional minor effect loci were reported on chromosomes 8 and 9, explaining up to 10% of phenotypic variance [15]. A QTL study based on high-throughput genotyping additionally identified other loci located in linkage groups 9 and 6, explaining 12 and 6% of phenotype variance [16]. These observations were in accordance with hypotheses on the effect of genetic background on the traits controlled by the *Ol* gene [17]. The genetic control of oleic acid accumulation thus remains under discussion.

In the present study, we analyzed two experimental crosses derived from genetically and phenotypically contrasted lines of the VNIIMK collection to map oleic acid content. To perform scanning for genetic markers for high OAC, we applied a genotyping by sequencing approach to obtain high-density SNP maps and next performed QTL mapping. The identified candidate SNP markers will be applied for the introduction of marker-associated selection (MAS) approaches with regard to sunflower breeding for oil resistance to thermal oxidation.

## Materials and methods

### Plant material used in the study

In the present study we used two independent F2 mapping populations (VK195xVK303 and VK876xVK101) derived from genetically contrasted lines of the VNIIMK collection that are currently used in the breeding process. The original Ol mutation was obtained by chemical mutagenesis in 1976 by chemical mutagenesis [9] this mutation was fixed in the variety called Pervenets, next this mutation was transferred in all high oleic lines from VNIIMK collection including VK195 and VK876. The parental plant material included genetically contrasting wild-type lines VK101 (*ol*/*ol*) and VK303 (*ol*/*ol*) expressing normal oleic phenotype (40–45%, of oleic acid). As mutant lines VK195 (*Ol/Ol*) and VK876 (*Ol*/*Ol*) express high oleic phenotype (>83% of oleic acid) were used. All lines were developed at the VNIIMK institute. Elite lines VK101 and VK303 are used to produce a simple interlinear middle-early sunflower hybrid “Typhoon” registered at VNIIMK [18]. Lines VK876 and VK195 are used to produce “Oxy” hybrid with high content of oleic acid and with γ- and δ-tocopherol forms that increase oxidative stability [6]. Male fertile forms of lines VK195 and VK876 were hand emasculated and were used as female parents. Fertile wild-type lines VK101 and VK303 were used as male lines to produce the pollen. The crosses were performed in the field (Krasnodar) in 2015. Individual F_1_ plants were selfed using isolators to produce the F_2_ progeny (F_2_ seeds) in the field (Krasnodar) in 2018. Seeds were obtained from a single inflorescence that was randomly selected from a plot.

### Phenotyping procedures

A total of 142 F_2_ seeds for cross VK195xVK303 and 144 F_2_ seeds for cross VK876xVK101 were assessed. According to the sunflower growth stages classification [19] seeds were harvested at the physiological maturity stage (R-9). A half-seed technique previously used to analyze the oil-related traits, including oleic acid content in sunflower was applied [20,21]. A single seed was cut transverse in half with a razor, and the first part containing the embryo was left for subsequent germination, DNA extraction and sequencing, while the second part was used for phenotyping. Oleic acid content was estimated by extracting fatty acids (FAs) and measuring FA composition. Half-seeds were homogenized and then subjected to fatty acid (FA) extraction with hexane followed by subsequent esterification as previously described [22]. The measurement was performed by means of Gas chromatography with flame ionization detection (GC-FID) using Chromateck-Crystal 5000 GC chromatograph with the DAG-2 M automatic dispensor. The percentage of each FA was calculated based on the peak area with the aid of the GS chromatography software. The relative content of oleic acid was used for QTL mapping. In addition to quantitative characterization, plants were distributed into high-oleic and non-high oleic categories. The relative content of 83% was used to distinguish between high-oleic and non-high oleic plants as previously described [23]. The phenotypic segregation ratio was tested using χ^2^ goodness-of-fit test. The phenotype data is represented in S1 Table.

### Genotyping procedures

To perform genotyping, half-seeds of sunflower lines left after cotyledon separation were germinated in rolls of filter paper, after which DNA was extracted from the cotyledon leaves using NucleoSpin Plant II kit (Macherey-Nagel) according to the manufacturer’s manual. GBS library was prepared according to the modified protocol [24] as described previously [25,26]. GBS library sequencing was performed in Illumina HiSeq 4000 with single-end reads with a length of 150 bp. Parental plants were genotyped in at least 7 biological replicates. Raw sequence data are available on NCBI SRA under the project number PRJNA742188. GBS sequencing barcodes are as previously described [27].

### SNP calling, genotype imputation, genetic map construction

SNP calling, genotype imputation, and genetic map construction were performed according to the methods previously described [27]. For cross VK876xVK101 the genetic map for chromosomes 7 and 14 was reconstructed as a putative potential translocation event in one parental line was identified between these linkage groups as a result of the cross. To reconstruct the genetic map for chromosomes 7 and 14, genetic markers that remained after filtering procedures (segregation distortion, proportion of missing genotypes) were used to compute linkage groups de novo using formLinkageGroups() function in r/qtl. Markers within each formed linkage group were ordered using the Kosambi mapping function. Additional filtering for SNPs within chromosomes was performed using the droponemarker() function; markers with LOD scores equal to or above −20 were discarded. For chromosomes with putative translocation, the chromosome that mostly contains genetic markers attributed to the 7th chromosome based on physical data (reference) was named as 7th. A similar rule was used for the 14th chromosome. The genetic map for the cross VK876xVK101 is attached as csv file.

### QTL mapping

Several approaches were applied in order to map oleic acid content. First, we applied simple interval mapping (SIM) adapted for non-normally distributed traits to map relative oleic acid content as well as SIM adapted for binary traits to map *Ol*-associated phenotype (high oleic trait). Additionally, composite interval mapping (CIM) was used to map the relative content of oleic acid. For QTL mapping, r/qtl package v1.50 [28] was used. The significance threshold was set using permutation analysis for the logarithm of odds (LOD) carried out with 1,000 iterations to find significant loci. A LOD value corresponding to the 99 percentile of the permuted LOD values distribution, was set as the significance threshold. To calculate the proportion of variance explained, analysis of variance (ANOVA) was applied within the R statistical package. LOD intervals for CIM were estimated using the lodint() function in r/qtl and 1.5-LOD confidence intervals were calculated with the expansion of the interval to the closest flanking marker. Phenotypic coefficient of variation (PCV), genotypic coefficient variation (GCV) as well as heritability (*h*^*2*^) were calculated for both crosses based on genotype and phenotype data.

## Results

### Oleic acid content assessment in studied crosses

**Fig 1:**
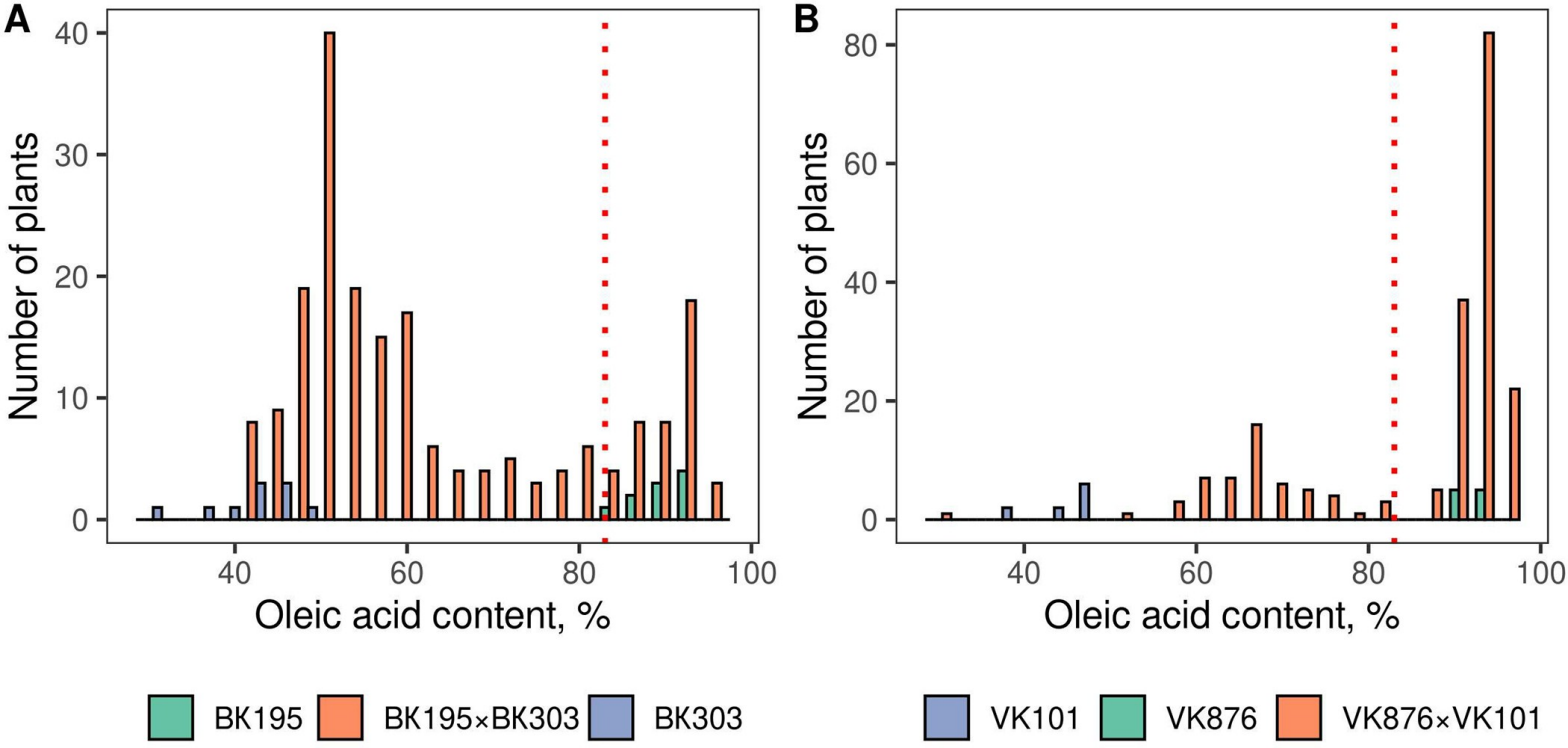
A histogram of the distribution of the relative oleic acid content for parental lines and F_2_ progeny. Panel A reflects the distribution of oleic acid content for cross VK195xVK303, panel B reflects the distribution of oleic acid content for cross VK876xVK101. The green color of the bars corresponds to high-oleic mutant parental lines, the blue color corresponds to low-oleic wild-type parental lines, and the orange color corresponds to F_2_ progeny. Dotted red vertical lines correspond to oleic acid content specific for high-oleic plants.

As a result of the phenotyping procedures the parental lines were characterized in terms of OAC in at least seven biological replicates. Regarding the progeny, 142 and 144 F_2_ seeds from the crosses VK195xVK303 and VK876xVK101 were characterized in terms of oleic acid content, respectively (S1 Table). For high oleic maternal lines VK195 and VK876 OAC was 89.95±3.16% and 90.82±1.4%, respectively, for non-high oleic lines VK303 and VK101 OAC was 40.25±4.8% and 45.2±4.76%. Bi-modal distribution of OAC in the progeny was specific for the two crosses (Fig 1). This could be explained by the fact that in the analyzed crosses high oleic trait is controlled by a single *Ol* gene which presence leads to the expression of the high oleic phenotype.

As monohybridism due to the single *Ol* gene was assumed, plants were distributed to high-oleic and non-high-oleic classes. As a result 104 and 40 F_2_ seeds from cross VK876xVK101 were classified as high-oleic and non-high-oleic, respectively. For cross VK195xVK303 28 and 114 F_2_ seeds from the cross were classified as high-oleic and non-high-oleic, respectively. Next χ^2^ goodness of fit was applied to test for monohybridism and expected 3:1 phenotype distributions (Table 1).

**Table 1:** The distribution of the F_2_ progeny into three high-oleic and one non-high oleic part.

Cross	High-oleic	Non-high-oleic	P-values for χ^2^ goodness of fit test for 3:1 segregation ratio
VK876xVK101	104	40	0.44
VK195xVK303	28	114	<2.2e-16

For the population VK876xVK101, the χ^2^ goodness of fit test confirmed the 3:1 segregation ratio (three high-oleic and one non-high-oleic), with the *Ol* allele behaving as dominant allele in that cross (Table 1). For VK195xVK303 χ2 goodness of fit test did not confirm the 3:1 segregation ratio (three high-oleic and one non-high-oleic). However in case if *Ol* allele is assumed to act as a recessive one for the population VK195xVK303, the χ^2^ goodness of fit test confirms a 1:3 segregation ratio (one high-oleic and three non-high-oleic), (P-value of χ^2^ goodness of fit test = 0.14).

### Genetic map reconstruction for chromosomes 7 and 14

**Fig 2:**
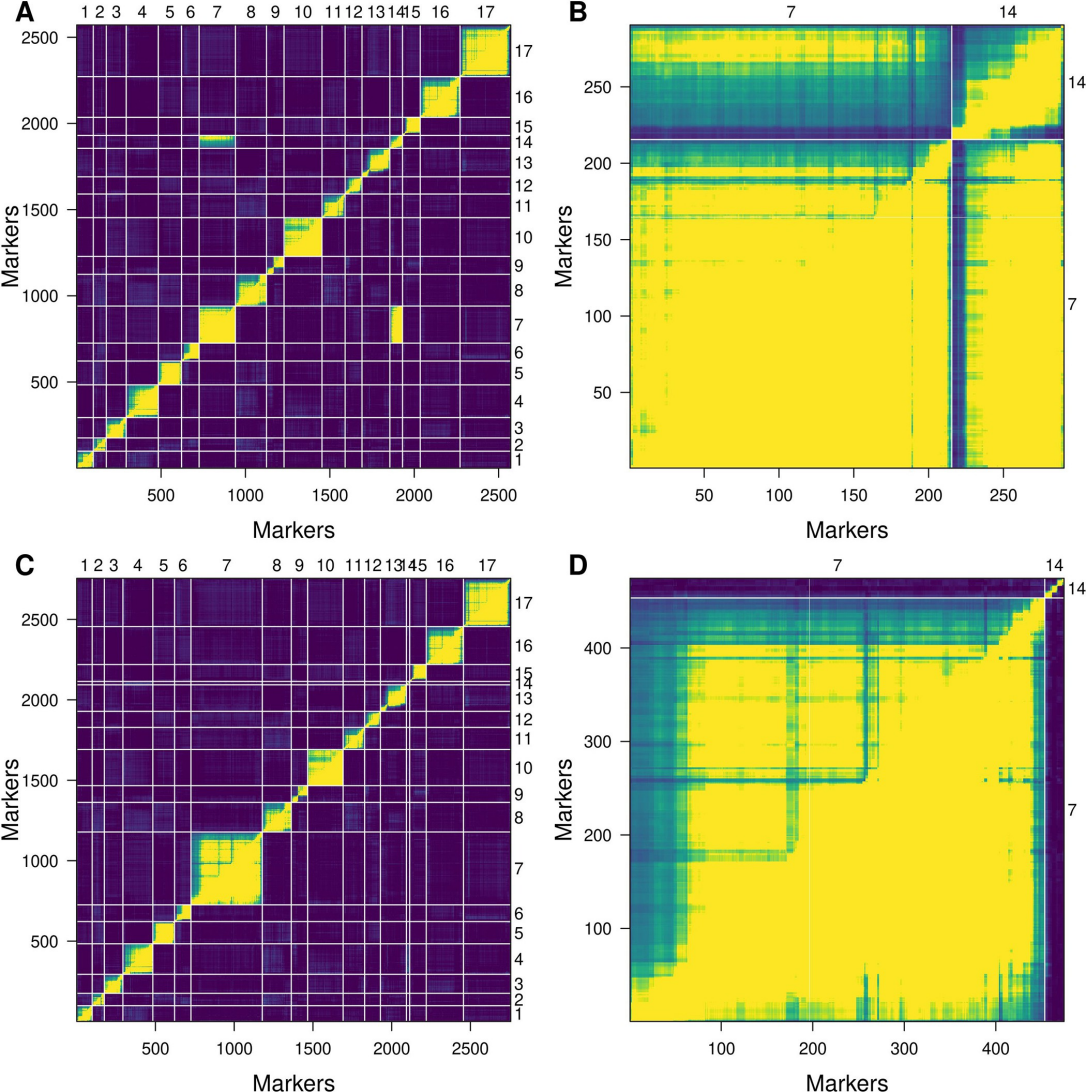
Reconstruction of the genetic maps for chromosomes 7 and 14 for cross VK876xVK101. Recombination fractions (upper triangle of the matrix) and LOD scores (lower triangle of the matrix) for the original genetic map of all chromosomes (Panel A) and chromosomes 7 and 14 (Panel B) and the reconstructed map for chromosomes 7 and 14 (Panel C an D).

Initially, independent genetic maps were constructed for the crosses based on the GBS data as previously described [27]. However, when QTL mapping for relative OAC was performed using the initial map for VK876xVK101, two peaks were identified on chromosomes 7 and 14 by simple interval mapping (S1 Fig). This was unexpected as it was supposed to identify a single locus. Detailed analysis of recombination fractions and LOD scores of the markers from chromosomes 7 and 14 revealed a tight linkage between genetic markers from these two linkage groups which could be a cause of identification of the second locus associated with OAC (Fig 2).Such tight linkage between the markers from chromosomes 7 and 14 could be the result of the potential translocation event and requires more studies in the future.

In order to fix these issues and map the locus of interest, genetic maps were reconstructed for chromosomes 7 and 14 from the genotyping data regardless of the information on the physical location of the markers according to genome assembly. This allowed removing the linkage between the two linkage groups. As a result, the size of the genetic map of chromosome 7 increased from 223.9 cM to 418.4 cM and the respective number of markers increased from 215 to 453 in the VK876xVK101 cross. The size of the 14th chromosome decreased from 127.9 cM to 111.3 cM, and the respective number of markers decreased from 75 to 21.

### Dissection of oleic acid content by QTL mapping

**Fig 3:**
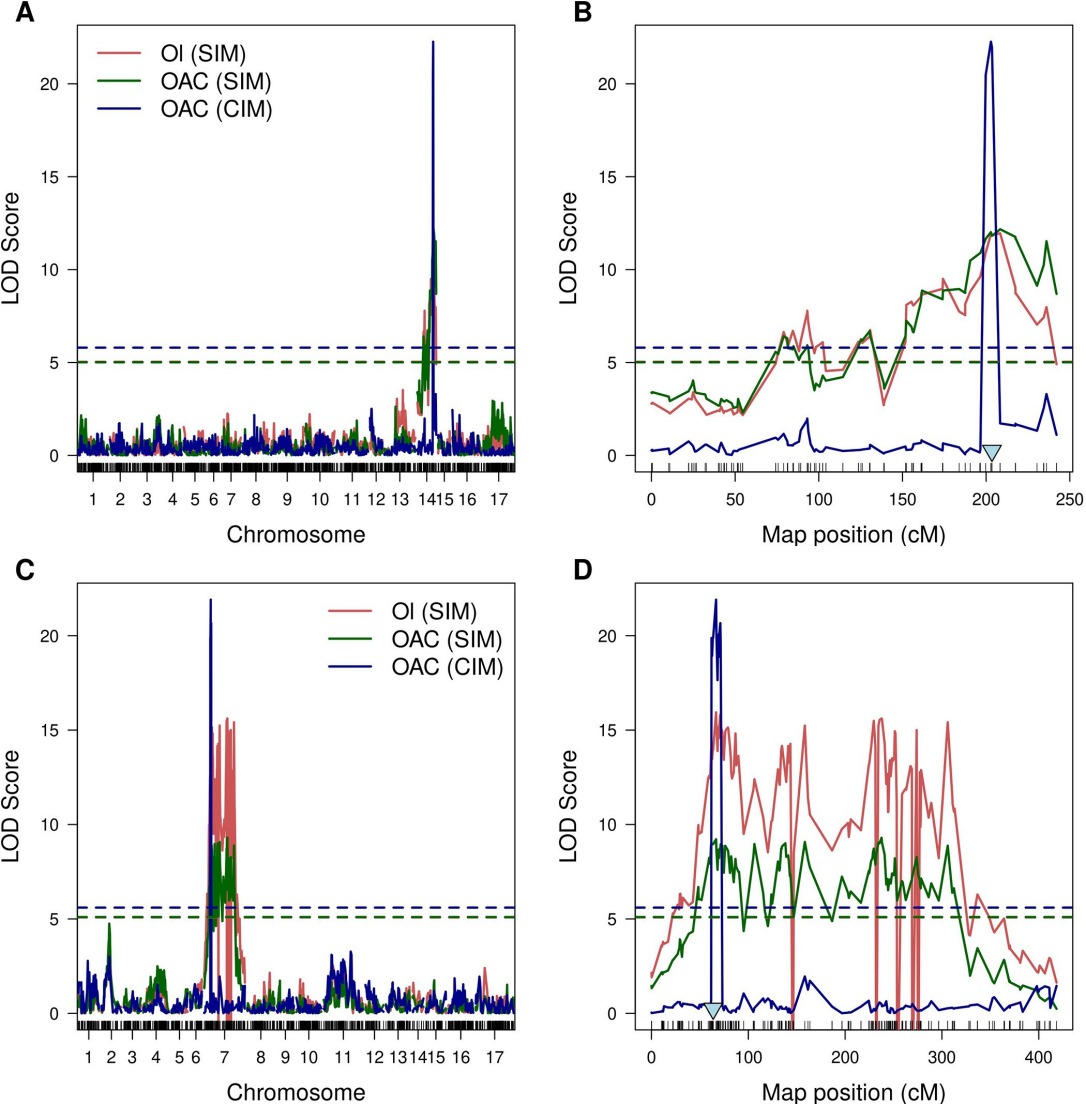
Likelihood curves of the LOD score *Ol* gene (high oleic trait) and oleic acid content (OAC). Results for cross VK195xVK303 represented at panels A and B and results for cross VK876xVK101 represented at panels C and D. The red curve reflects the results of mapping the binary trait associated with the presence and/or absence of the *Ol* gene with the interval mapping approach. The green curve reflects the results of interval mapping of OAC adapted for non-normally distributed traits. The blue curve reflects the results of composite interval mapping of oleic acid content. Colored horizontal dashed lines correspond to the permutation threshold for respective mapping approaches. Light blue triangles indicate markers that are most closely located to the *Ol* gene encoding FAD2-1 based on the physical map data.

After phenotype data were collected and genetic maps were constructed, we performed the mapping of oleic acid content (S2 Table). Additionally having information on genotype and phenotype data, phenotypic coefficient of variation (PCV), genotypic coefficient variation (GCV) as well as heritability (*h*^*2*^). PCV, GCV and *h*^*2*^ equaled 25.3%, 22.2% and 76.4%, respectively, for cross VK195xVK303. For cross VK876xVK101 PCV, GCV and *h*^*2*^ equalled 15.5%, 11.1% and 50.5% respectively. For trait mapping, we applied SIM in order to map OAC as a quantitative and qualitative trait. Both approaches identified wide peaks on chromosome 14 for the cross VK195xVK303 and chromosome 7 for the cross VK876xVK101 (Fig 3, S2 Table). As the identified intervals were large, a more conservative CIM approach was used in order to narrow the associated regions. Next, 1.5-LOD confidence interval was calculated for each of the cross to identify potential genetic markers.

For the cross VK195xVK303, the 1.5-LOD confidence interval calculated for CIM results spanned from 199.76 to 208.4 cM. According to the physical map information, a genetic marker S14_137961667 was most closely (13.3 megabases away) located to the gene encoding FAD2-1 (NCBI GeneID 110904312) located from 151341021 to 151344179 bp and associated with high OAC [14,29]. It also should be noted that based on the interval mapping (IM) results the closest significant marker (S14_154333404, IM LOD score: 9.13) was located 2.9 megabases away (S2 Table). For the cross VK876xVK101, the 1.5-LOD confidence interval obtained for significant markers identified by composite interval mapping from chromosome 7 spanned from 63.72 to 71.45 cM. This interval included one marker S14_78139380 which should be located on chromosome 14 based on the physical map (Table 2). As it is derived from the 14th chromosome we checked the physical location of this marker and it was demonstrated that S14_78139380 was located 73 Mb away from the FAD2-1 gene. Such a big distance could be explained by the fact that genetic markers located close to the FAD2-1 gene based on the physical map were filtered out during genetic map construction for cross VK876xVK101.

**Table 2:** Summary of genetic markers located within 1.5-LOD confidence interval resulted from composite interval mapping of OAC.

Marker	Cross	Chromosome	Position (cM)	mutant allele (A)	wt allele (B)	LOD score	PVE, %
S14_136819253	VK195 x VK303	14	199.76	A	G	20.48	53.95
S14_137821336		14	202.86	G	C	22.27	56.37
S14_137795880		14	202.86	T	C	22.27	55.73
S14_137889873		14	203.49	A	G	21.96	56.07
S14_137951402		14	203.49	A	T	21.96	56.18
S14_137961667		14	203.49	G	A	21.96	56.41
S14_138978611		14	208.4	A	G	1.72	54.86
S14_78139380	VK876 x VK101	7	63.72	G	A	19.88	46.99
S7_37821562		7	66.74	G	A	21.91	53.29
S7_39112905		7	66.74	A	G	21.91	50.46
S7_39776264		7	67.11	T	A	20.03	44.27
S7_39681220		7	67.11	A	G	20.03	47.39
S7_25411825		7	68.23	G	A	17.65	48.38
S7_25624783		7	68.23	G	A	17.65	46.14
S7_25717162		7	68.23	G	A	17.65	44.37
S7_25624784		7	68.23	G	A	17.65	46.14
S7_25664488		7	68.23	G	A	17.65	46.24
S7_25624756		7	68.23	A	G	17.65	46.14
S7_25421436		7	68.23	C	T	17.65	46.46
S7_25726978		7	68.23	C	A	17.65	44.37
S7_76273882		7	68.92	T	C	19.98	49.05
S7_25876036		7	68.92	A	G	19.98	48.62
S7_76319288		7	68.92	G	A	19.98	49.05
S7_25964564		7	68.92	C	T	19.98	48.83
S7_75968410		7	68.92	C	T	19.98	48.91
S7_76547907		7	68.92	A	C	19.98	49.11
S7_25961858		7	68.92	A	C	19.98	48.75
S7_25855919		7	68.92	T	G	19.98	48.62
S7_75140370		7	69.28	C	A	20.07	48.45
S7_72038884		7	69.28	T	C	20.07	51.02
S7_75826681		7	69.64	G	A	20.08	49.09
S7_75503792		7	69.83	A	T	18.88	46.9
S7_75503823		7	69.83	C	T	18.88	46.9
S7_75934390		7	70.02	G	A	20.08	49.01
S7_75892237		7	70.02	T	A	20.08	49.09
S7_75894251		7	70.02	G	A	20.08	49.09
S7_75535778		7	70.02	C	T	20.08	49.02
S7_76736113		7	71.1	T	C	20.66	50.33
S7_76758370		7	71.1	T	A	20.66	50.33
S7_76852814		7	71.45	T	C	19.73	48.72

Values were rounded to the second digit.

**Fig 4:**
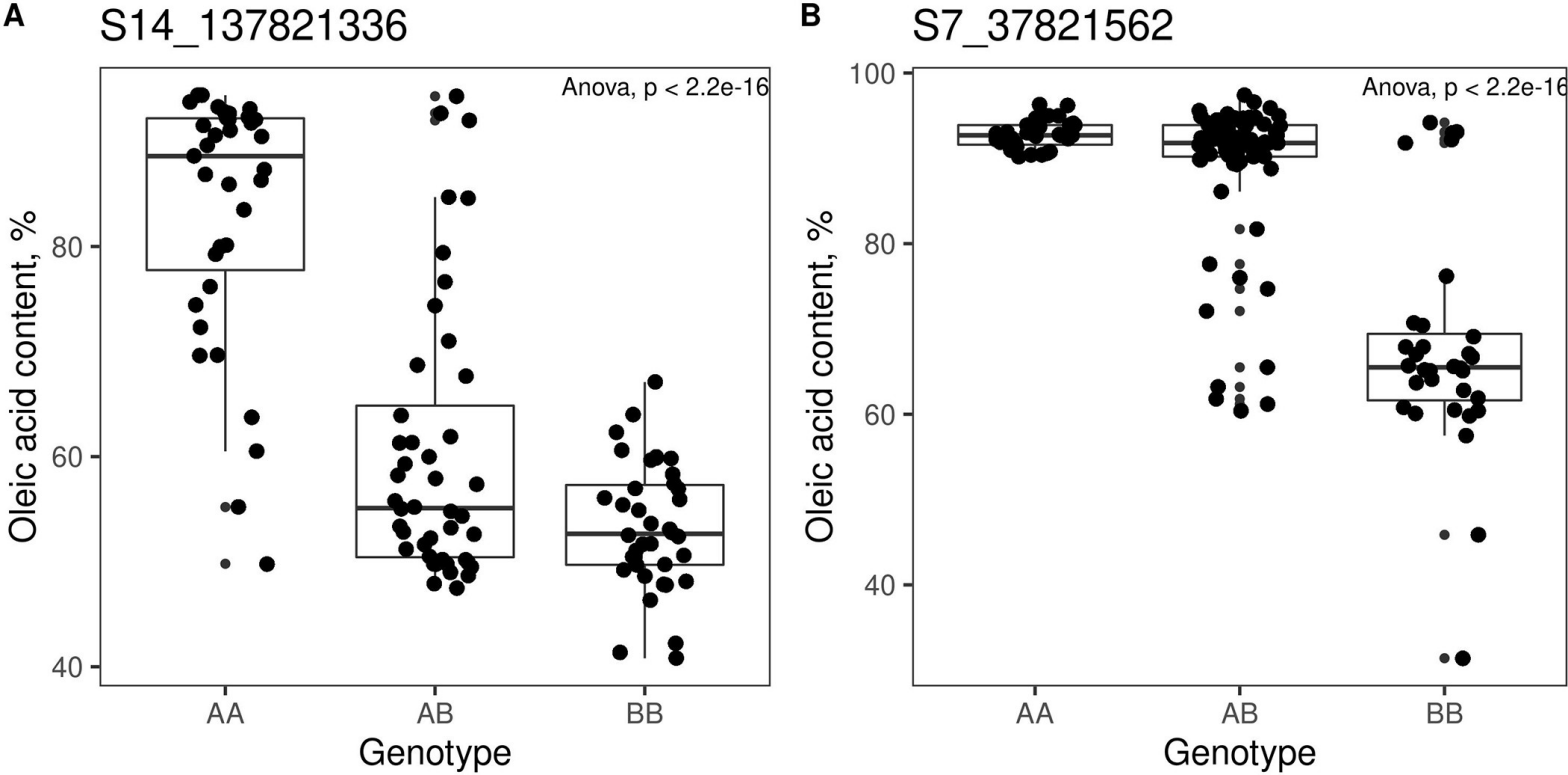
Boxplots for genotype effects on oleic acid content. AA–genotype corresponds to the maternal (mutant) line, BB–genotype corresponds to the wild-type male line. Panel A reflects the genotype effect of the most significant marker S14_137821336 for the cross VK195xVK303. Panel B reflects the genotype effect of the most significant marker S7_37821562 for the cross VK876xVK101.

The proportion of variance explained (PVE) was calculated for 1.5-LOD confidence intervals of CIM (Table 2). For the cross VK195xVK303 the maximum PVE was as high as 56.37% while for the cross VK876xVK101 maximum PVE equaled to 53.29% (Table 2). Additionally, visualization of the genotype effects of the markers with the highest LOD score demonstrated that markers from cross VK876xVK101 and VK195xVK303 have different, dominant/recessive effects on phenotype distribution (Fig 4).

For the cross VK195xVK303, genetic markers for high content of oleic acid demonstrated a recessive nature (Fig 4), which was expected taking into account the analysis of phenotype distribution that gave the opposite result compared to the expected 3:1 segregation ratio, where 3 should correspond to high-oleic phenotypes and 1 non-high oleic phenotypes. For the cross VK876xVK101, genetic markers for high content of oleic acid demonstrated dominance as it was demonstrated previously for *Ol* gene-associated markers.

## Discussion

The present study aimed to identify potential genetic markers for high oleic acid content in sunflower. To do so, we performed phenotyping and genotyping of the two experimental crosses derived from parental lines contrasting in OAC. Genotyping was performed using the genotyping-by-sequencing approach. Based on the obtained data we reconstructed high-density SNP maps and identified genetic markers by means of QTL mapping for the studied trait.

Our results support the previously discussed hypothesis on the monogenic control of the high oleic acid trait [10,13,29]. Despite several studies discussing the role of the additional genes that affect major effects, no additional loci were identified for both of the studied crosses in the present study (Fig 3). Genetic markers for both of the crosses demonstrated a strong effect on the phenotype and explained up to 56% of phenotypic variation. A similar proportion of variance (56–86%) was explained by the genetic markers previously identified for oleic acid content associated with *Ol*-gene [13,15,29]. Notably, both loci identified contained the genetic markers located close to FAD2-1 gene (*Ol*) based on the physical map information. Our experimental design based on F_2_ segregation is however not the best suited to identify minor QTLs due to a low power of phenotype evaluations.

We identified that *Ol*-associated markers had two different modes of inheritance (recessive/dominant) in the two studied crosses. At the level of F_2_ progeny phenotype distribution, the crosses VK195xVK303 and VK876xVK101 demonstrated opposite patterns of intralocus relationships (Fig 1), yet consistent with monohybridism. These observations were supported by the QTL analysis revealing the genetic effects of markers. Namely, for the cross VK876xVK101 genetic markers proved to be dominant, while for the cross VK195xVK303 genetic markers appeared to be recessive, which was not the case with the previously reported markers (Fig 4).

The dominant nature of the *Ol* mutation is explained by the complex organization of the locus containing two copies of genes encoding FAD2-1 [14]. It was demonstrated that the presence of a second reduced copy leads to the accumulation of the small interfering RNAs that induce gene silencing of the first functional copy [23]. Thus the single allele of *Ol* gene is enough to cause high OAC [23,29]. Despite the clear evidence of the dominant nature of *Ol* that was demonstrated, recessive behavior of the *Ol* gene was sometimes described when crossing high oleic lines with non-high oleic lines [17,30]. Such recessive *Ol* behavior of *Ol* mutation was hypothesized by the presence of an *Ml* allele of the putative modifier gene which converts *Ol* into a recessive state [12]. The presence of a putative suppressor gene was also suggested when obtaining 3:1 (non-high-oleic:high-oleic) while analyzing segregation populations of sunflower [31]. It is also possible to hypothesize about the presence or appearance of the additional "recessive" *Ol* allele in VK195, via emergence of novel mutation.

In addition, we also identified a potential translocation event between chromosomes 7 and 14 in the cross VK876xVK101. Initially maps for crosses VK195xVK303 and VK876xVK101 were based on the genomic information i.e. physical location of the genetic markers [27], a similar method was used to construct maps for mungbean and sorghum [32,33]. Possible translocation was identified based on the linkage found between the genetic markers from chromosome 7 and 14, as well as due to the association of markers physically located on chromosome 7 with the OAC was first reported in the present study. Due to this potential translocation, we reassembled the maps for chromosomes 7 and 14 without previous information on the physical location of the genetic markers for these chromosomes as it was previously done for barley and yellow alfalfa [34,35]. Notably, it was previously demonstrated that *Ol*-associated markers are located on chromosome 14 [13–15,23]. Previously translocations were discussed for *Helianthus* genus in terms of divergence between the species, namely *H*. *annuus* and *H*. *argophyllus* were hypothesized to differ by two reciprocal translocations [36,37], with five non-reciprocal translocations between the chromosomes of *H*. *annuus* and *H*. *argophyllus* having been additionally reported [38]. Eight translocations were discussed among the differences between *H*. *annuus* and *H*. *petiolaris* [39]. Additionally, greater divergence in terms of translocations was reported between *H*. *annuus* and *H*. *niveus* totaling in the identification of 18 translocated segments [40]. Although the translocation events were reported between *H*. *annuus* and some of other *Heliathus* species, no translocations were reported within *H*. *annuus*. Hence, our study is among the first to report this potential event.

Thus, the results of the present study on the one hand allowed us to identify genetic markers associated with strong effects on the oleic acid content that explain up to 56% of phenotypic variation. These markers will be used for marker-assisted breeding of sunflower for oil quality. On the other hand, we observed different states for the markers (dominant/recessive) identified from different crosses. This observation could serve as the basis for further studies on modificator gene and/or recessive allele identification in the lines VK195 and VK303. Additionally, a possible chromosomal translocation has been identified between chromosomes 7 and 14. This observation in turn could serve as a basis for application of the cytogenetics approaches to study the genome structure of the lines VK876 and VK101 to more detail.

## Supporting information

S1 Fig Likelihood curves of the LOD score *Ol* gene (high oleic trait) and oleic acid content (OAC) for VK876xVK101 (panels A and B) crosses. The mapping was performed using original genetic map for VK876xVK101 cross [27]. The red curve reflects the results of SIM for the binary trait associated with the presence and/or absence of the *Ol* gene with the interval mapping approach. The green curve reflects the results of SIM adapted for non-normally distributed traits of OAC. The blue curve reflects the results of CIM of OAC. Colored horizontal dashed lines correspond to the permutation threshold for respective mapping approaches. Light blue triangle indicate marker that is most closely located to the *Ol* gene encoding FAD2-1 based on the physical map data.
(TIFF)

S1 Table Summary table on phenotype data. (XLS)

S2 Table Results of CIM and SIM for all markers. (XLS)

S1 File Genetic map for cross VK876xVK101. (CSV)
